# Reaching Those At Risk for Psychiatric Disorders and Suicidal Ideation: Facebook Advertisements to Recruit Military Veterans

**DOI:** 10.2196/10078

**Published:** 2018-07-05

**Authors:** Alan R Teo, Samuel BL Liebow, Benjamin Chan, Steven K Dobscha, Amanda L Graham

**Affiliations:** ^1^ HSR&D Center to Improve Veteran Involvement in Care (CIVIC) VA Portland Health Care System Portland, OR United States; ^2^ Department of Psychiatry Oregon Health & Science University Portland, OR United States; ^3^ School of Public Health Oregon Health & Science University and Portland State University Portland, OR United States; ^4^ Schroeder Institute for Tobacco Research & Policy Studies Truth Initiative Washington, DC United States; ^5^ Department of Oncology Georgetown University Medical Center Washington, DC United States

**Keywords:** Facebook, social media, methodology, Veterans Affairs, veterans

## Abstract

**Background:**

Younger military veterans are at high risk for psychiatric disorders and suicide. Reaching and engaging veterans in mental health care and research is challenging. Social media platforms may be an effective channel to connect with veterans.

**Objective:**

This study tested the effectiveness of Facebook advertisements in reaching and recruiting Iraq and Afghanistan-era military veterans in a research study focused on mental health.

**Methods:**

Facebook ads requesting participation in an online health survey ran for six weeks in 2017. Ads varied imagery and headlines. Validated instruments were used to screen for psychiatric disorders and suicidality. Outcomes included impressions, click-through rate, survey completion, and cost per survey completed.

**Results:**

Advertisements produced 827,918 impressions, 9,527 clicks, and 587 survey completions. Lack of enrollment in Veterans Affairs health care (193/587, 33%) and positive screens for current mental health problems were common, including posttraumatic stress disorder (266/585, 45%), problematic drinking (243/584, 42%), major depression (164/586, 28%), and suicidality (132/585, 23%). Approximately half of the survey participants (285/587, 49%) were recruited with just 2 of the 15 ads, which showed soldiers marching tied to an “incentive” or “sharing” headline. These 2 ads were also the most cost-effective, at US $4.88 and US $5.90 per participant, respectively. Among veterans with current suicidal ideation, the survey-taking image resulted in higher survey completion than the soldiers marching image (*P*=.007).

**Conclusions:**

Facebook advertisements are effective in rapidly and inexpensively reaching military veterans, including those at risk for mental health problems and suicidality, and those not receiving Veterans Affairs health care. Advertisement image and headlines may help optimize the effectiveness of advertisements for specific subgroups.

## Introduction

Military veterans who served during the Iraq and Afghanistan conflicts are at an elevated risk for a number of psychiatric problems. In a Veterans Affairs (VA) sample, one in four were found to have at least one mental health diagnosis, mostly commonly posttraumatic stress disorder (PTSD), depression, and alcohol and substance use disorders [1]. Heightened risk for suicide is also a major concern [2], with rates of suicide among veterans in the United States markedly higher than the general population, even after adjustment for age and gender differences [3]. Despite this, approximately 40% of Iraq and Afghanistan veterans have never accessed VA health services [4], and even when these veterans are in VA care, they may not be more inclined to utilize mental health care if experiencing active suicidal ideation [5].

Preventing veteran suicide has been, and continues to be, a top priority. Nonetheless, in-person health care appointments pose a significant barrier to Iraq and Afghanistan era veterans who tend to be younger and more likely to be employed than other veterans [6,7]. Other common barriers to formal help-seeking and treatment access, even in the presence of seemingly severe symptoms such as suicidal ideation or behaviors, include low perceived need [8-10], distance from health care facilities [11], and a desire to “handle the problem alone” [8]. In a sample of veterans who died by suicide in Oregon, an estimated 78% had not accessed VA health services [12].

Traditional strategies for recruiting participants into research studies can suffer from narrow reach, geographical limitations, costliness, and time-intensiveness. By comparison, recruitment via social media platforms, especially Facebook, may be faster, cheaper, and easier than traditional methods [13–15]. Among social media platforms, Facebook may be an especially important tool because it is the largest and most used, with a diverse base of users with detailed demographic profiles [13]. Facebook users spend upwards of 50 minutes a day on the platform, and among online adults between the ages of 18 to 29, approximately 9 of 10 use Facebook [14,15].

In recent years, many studies have examined Facebook advertisements (ads) as a recruitment method for research studies on mental or behavioral health. Facebook ads have been used effectively in populations including college students and young adults [16,17] and military veterans particularly those with risky drinking [20–23]. Prior research has also suggested ad campaigns can achieve both “broad reach and targeted recruitment,” and found ad costs to be manageable [18]. Throughout this paper, we use the term “reach” to mean even a minimal amount of engagement with an ad, not necessarily engagement in health care, a definition that is consistent with the vein in which it is used in social media contexts. Nonetheless, much work remains in the development of best practices and evidence-based recruitment strategies on social media. Studies conducting experiments comparing particular Facebook advertising approaches, such as differing images or text are lacking [19,21,25–27]. One recent study by Pedersen and colleagues, which focused on recruiting spouses of heavy drinking service members and veterans, did systematically and sequentially test different ad features. It concluded that ads with text accentuating the US $120 financial incentive for study participation had a higher conversion to study participation at a lower per-participant cost [19]. Examination of a spectrum of potential outcomes, ranging from general exposure (eg, “impressions”) to initial interaction (eg, link clicks) to implementation (eg, enrollment in a research study) [20], would also be helpful in social media studies.

We conducted a study with three aims: (1) determine the feasibility of reaching Iraq and Afghanistan era military veterans through Facebook ads, (2) quantify the extent to which reached veterans are at risk for psychiatric problems, and (3) characterize how veterans utilize social media and interact with their social networks on Facebook. In this article, we focus on the first two aims. More specifically, we determined the recruitment of military veterans to a mental health focused research study, examined what ad features are most relevant to engaging veterans, and characterized what kinds of veterans are likely to be reached by the ads. As an exploratory study, we limited our a priori hypothesis to state that it would be feasible to recruit recent military veterans with probable mental health problems.

## Methods

### Participants

The target population for the survey was United States (US) military veterans of the Operation Enduring Freedom-Operation Iraqi Freedom (OEF-OIF) service era (September 2001-present), also referred to as Iraq and Afghanistan era veterans. To be eligible, individuals needed to be age 18 or older, and on active duty in the US Armed Forces after September 2001 but not presently. We excluded individuals who completed surveys in less than five minutes, had more than one survey response, or incorrectly answered a military-related “insider knowledge” question to reduce the chance of online survey misrepresentation [18,21]. Survey completion was defined as those respondents who reached the end of the online survey and were not excluded based on the above quality control measures.

### Advertisement Campaign

Facebook offers myriad options related to placement and targeting of ads; the same parameters were used for all ads. Ads were run simultaneously, to identical audiences, with the same ad budget, and for the same duration of time. Ads ran for a total of 45 days between January 13, 2017, and March 18, 2017, except for one ad that was briefly deactivated by Facebook for technical reasons. Ads were exclusively placed in the News Feed on computers and not mobile phones as the survey was developed for computer administration. Ads were optimized per Facebook’s algorithm for clicks, meaning that ads were automatically shown to users whom Facebook anticipated would click at the highest rates, in a targeting process adjusted by actual clicks during the campaign.

Study ads were broadly targeted at Facebook users in the US of any age or gender who had at least one of a variety of veteran-related characteristics (eg, interest in “United States Armed Forces” or “Supporting Our Veterans” as determined by their Facebook profile). Text above the ad image indicated that veterans who served between 2001 and 2017 were needed for an “online health survey.”

**Figure 1:**
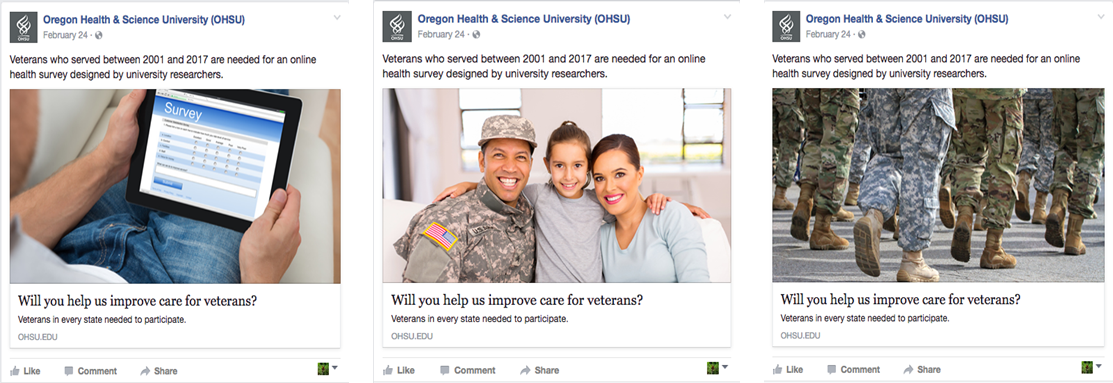
Sample Facebook ads illustrating the three different ad images (Survey-Taking, Family, and Soldiers Marching).

We designed a total of 15 ad variations in a 3x5 factorial design, with 3 images (ie, a person taking a survey on a tablet device; a veteran with his family; and soldiers marching) varied against five headlines. Ad images are illustrated in Figure 1. Headlines were informed by empirical research in psychology and survey methodology as well as established principles in behavioral economics known to help nudge behavior [22].

Specific approaches that can motivate research participation include: targeting feelings of altruism or a “warm-glow” effect [23], harnessing “psychological capital”, which is closely correlated with a sense of empowerment [24,25], or using a statement of what others in similar situations do, also known as descriptive social norms [26]. Providing financial incentives [27] or encouraging the sharing of content with social network members could also increase engagement [28]. Based on these ideas, we crafted and used the following 5 ad headlines:

1. *Altruism*: “Will you help us improve care for veterans?”
2. *Empowerment*: “You can tell us how to design new health programs for veterans.”
3. *Social norms*: “Hundreds of veterans are participating in this survey. Will you join them?”
4. *Incentive*: “You can win a new 7.9” 16 GB iPad Mini 4 with Retina Display!”
5. *Sharing*: “Will you share this with one veteran you know?”

Ads were hosted by Oregon Health & Science University (OHSU) and linked to an online survey. To calculate survey participation and other outcomes by an ad, we constructed separate uniform resource locators (URLs) to the online survey for each ad. Prospective participants initially completed an online consent and eligibility screener. As an incentive for survey completion, we informed potential participants of an optional sweepstakes or lottery, in which two randomly selected survey participants who provided contact information would receive an iPad. Eligible, consented participants then completed the full online survey. Before and after survey, we provided all participants with a series of online, phone, and text messaging mental health treatment resources, including options for crisis situations, non-urgent treatment referral, and—as we would not be aware of the particular location of respondents—ways to locate local support and treatment resources. The institutional review board of OHSU approved all study procedures.

### Measures

Our primary outcome was survey completion, which represents the highest level of engagement with a Facebook ad [20]. As additional outcomes, we included measures of ad engagement that are automatically tabulated by Facebook for advertisers:

- *Impressions*: the total number of times that the ad is presented to any Facebook user.
- *Clicks*: the number of times that a user clicks on the ad.
- *Click-through rate (CTR)*: the number of clicks divided by impressions.
- *Reactions*: the total number of “Likes” or other Facebook reactions (“Love,” “Haha,” “Wow,” “Sad,” and “Angry”) generated by an ad.

The online survey included a series of self-report questions to obtain background information about the sample including sociodemographic characteristics, military history, social media use, and interest in social media-based interventions. Using survey items from the National Survey of Veterans [29], we assessed the period of service (ie, “Have you ever served on active duty in the US Armed Forces? *Active duty includes serving in the US Armed Forces as well as activation from the Reserves or National Guard.” and “* When did you serve on active duty in the US. Armed Forces? *Mark all service eras that apply.* *”*), branch of service (ie, “In which branch or branches did you serve on active duty?”) and deployment history (ie, “Did you deploy in support of Operation Enduring Freedom (OEF) or Operation Iraqi Freedom (OIF) or Operation New Dawn (OND)”) Frequency of Facebook use was assessed by adapting previously validated survey items used by Pew Research [30]. We used two additional items from the National Survey of Veterans to determine VA health service use (ie, “Have you ever been enrolled in VA health care?” and “In the past 12 months, did you use any VA health care services?”) [29]. Participants who responded “Don’t know” to health service use questions were classified as not non-users.

To screen for mental health problems, we employed reliable and valid self-report tools. For PTSD, we used the Primary Care PTSD Screen for DSM-5 (PC-PTSD), a five-item scale assessing past-month symptoms of a lifetime traumatic event. A score of three or higher on the PC-PTSD indicates a positive screen [31]. For alcohol misuse, we used the Alcohol Use Disorders Identification Test Alcohol Consumption Questions (AUDIT-C), a three-item scale on frequency and intensity of drinking. An AUDIT-C score of four or higher for men, or three or higher for women, indicates a positive screen for problematic drinking [32]. For major depression, we used the Patient Health Questionnaire-2 (PHQ-2), a two-item scale on anhedonia and depressed mood in the previous two weeks. A score of two or higher on the PHQ-2 indicates a positive screen [33]. For suicidality, we used the Depressive Symptom Inventory Suicidality Subscale (DSI-SS), a four-item scale on suicidal ideation within the past two weeks [34]. A score of two or higher on the DSI-SS indicates a positive screen in a population-based sample [35].

### Statistical Analysis

Demographic variables were compared by ad text and image for participants in the analytic sample using Pearson’s chi-square test, or ANOVA for age. All outcomes were modeled as negative binomial counts. The study design parameters of image, headline, and the interaction were included as independent factors. The model for clicks and CTR included an offset for the number of impressions; the model for reactions included an offset for the number of clicks.

## Results

### Feasibility of Recruiting Military Veterans Through Facebook Advertisements

**Figure 2:**
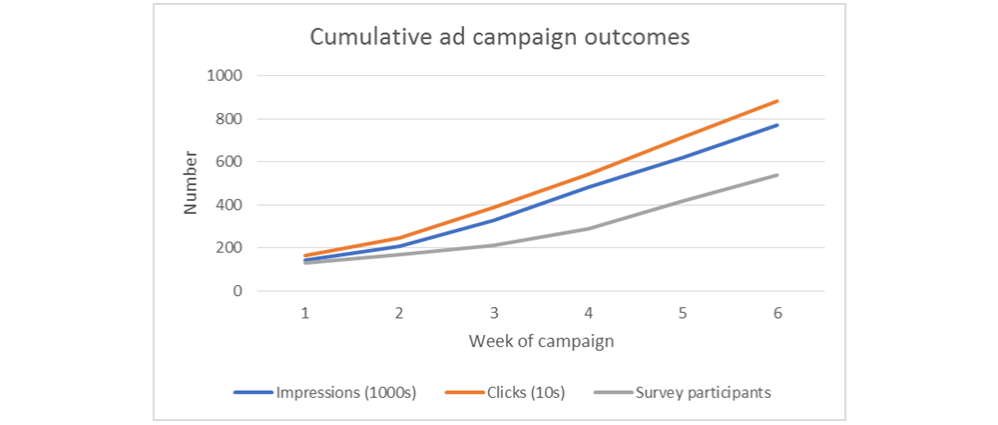
Cumulative ad campaign outcomes over time.

Over the 45 days of the advertising campaign (Figure 2), the Facebook ads produced 827,918 impressions, 9,527 clicks (CTR=9,527/827,918, 1.20%), and 1,787 reactions. There were 1,329 complete responses to the eligibility screener, of which 711 met eligibility criteria, and 605 completions of the online survey (ie, 605/711, 85% response rate). A total of 18 responses were excluded from the analysis based on quality control measures. Ten took less than 5 minutes, 2 claimed nonexistent pay grades, and 6 were duplicate responses. This left a final sample of 587 (ie, roughly 13 new participants each day). Total ad expenditure was US $11,427, yielding an average cost per analyzed survey of US $19.47.

### Characteristics of Recruited Veterans

Characteristics of survey participants are described in Table 1. Their mean age was 40 years. A total of 81% (474/587) were male, and 81% (477/587) were white and non-Hispanic. In addition to serving during the Iraq and Afghanistan era, many also served during prior eras, particularly the Gulf War era (213/587, 36%). The majority (426/587, 73%) had been deployed in support of OEF-OIF. In this sample, the majority (326/587, 56%) were in the Army, as compared with approximately 36% among active-duty military personnel [36].

With regards to VA enrollment, 33% of participants (193/587) had not enrolled in VA health services and 55% (322/587) had not used VA care in the prior year. Positive screens for current mental health problems were common: PTSD (266/585, 45%), problematic drinking (243/584, 42%), and major depression (164/586, 28%). Twenty-three percent (132/585) indicated current suicidality. Of the participants not enrolled in VA health services, 21% (40/193) reported current suicidality.

### Associations Between Advertisement Characteristics and Demographic Characteristics

Gender of respondents varied by ad text, (χ^2^_4_ (N=583)=10.7, *P*=.03), with *sharing* and *empowerment* messages having a higher proportion of women. Age varied by text (F(2, 585)=11.84, *P*<.01) and image (F(2, 585)=10.09, *P*<.01), with the *soldiers marching* image and *incentive* text attracting the youngest respondents and the *survey-taking image* and *altruism* and *social norms* headlines attracting the oldest. Service era varied correspondingly, with the *soldiers marching* image (χ^2^_2_ (N=585)=9.90, *P*<.01) and *incentive* text (χ^2^_4_, N=585)=22.03, *P*<.01) attracting a higher proportion of respondents who had only served during the OEF-OIF era. Race, ethnicity, education, military branch, and deployment to Iraq or Afghanistan did not vary significantly by ad text or image.

### Variations in Advertisement Engagement and Cost

There was a main effect for ad image across impressions, CTR, and reactions, but not survey participation. In terms of both impressions and click-through rates, the *soldiers marching* image performed better than the *survey-taking* and *family* images (*P*<.001 for all comparisons). In addition, the *soldiers marching* image generated significantly more reactions than the *survey-taking* (*P*=.001) and *family* (*P*<.001) images. However, there were no significant differences by ad image in terms of survey participation.

**Table 1:** Descriptive characteristics of all survey participants (N=587).

Characteristic		Value
**Demographics and Military History**			
	Age in years, mean (SD)	40.0 (12.0)
	Male, n (%)	474 (80.8)
	Racial or ethnic minority, n (%)	110 (18.9)
**Branch of military service^a^, n (%)**		
	Army	326 (55.5)
	Navy	110 (18.7)
	Air Force	109 (18.6)
	Marine Corps	75 (12.8)
	Coast Guard	11 (1.9)
	Other	2 (0.3)
**Service era^b^, n (%)**			
	September 2001-present (includes Iraq and Afghanistan conflicts)	587 (100)
	August 1990-August 2001 (includes Gulf War)	213 (36.3)
	May 1975-July 1990	112 (19.1)
	August 1964-April 1975 (includes Vietnam era)	29 (4.9)
	Deployed to Iraq or Afghanistan	426 (72.7)
**Education, n (%)**			
	High school diploma or less	34 (5.8)
	Some college, or vocational degree	250 (42.6)
	College degree or greater	303 (51.6)
**Marital status, n (%)**			
	Single, never married	112 (19.1)
	Divorced, separated, or widowed	111 (18.9)
	Married or living as married	363 (62.0)
**Facebook use frequency, n (%)**			
	Every few weeks or less often	14 (2.4)
	Weekly or a few times a week	47 (8.0)
	Daily or more often	524 (89.6)
**Clinical characteristics, n (%)**			
	Positive depression screener^c^	164 (28.0)
	Positive posttraumatic stress disorder screener^d^	266 (45.5)
	Positive alcohol misuse screener^e^	243 (41.6)
	Positive suicidal ideation screener^f^	132 (22.6)
**Veterans Affairs Health Service Use, n (%)**			
	Not enrolled	193 (32.9)
	Not used in last year	322 (54.9)

^a^Percentages do not add up to 100% because some respondents (45/587, 7.7%) indicated multiple branches.

^b^Percentages do not add up to 100% because some respondents indicated multiple service eras; 60.5% (355) served only during the current era, September 2001 to present.

^c^Patient Health Questionnaire-2 (PHQ-2) score ≥ 3. Due to missing item response, the total number of respondents for this scale was 586.

^d^Primary Care posttraumatic stress disorder (PTSD) Screen for DSM-5 (PC-PTSD-5) score ≥ 3. Due to missing item response, the total number of respondents for this scale was 502.

^e^Alcohol Use Disorders Identification Test Alcohol Consumption Questions (AUDIT-C) score ≥ 4 (men) or ≥ 3 (women). Due to missing item response, the total number of respondents for this scale was 477.

^f^Depressive Symptom Inventory Suicidality Subscale (DSI-SS) score ≥ 2. Due to missing item response, the total number of respondents for this scale was 585.

**Table 2:** Matrix of 15 Facebook advertisement variants with outcomes for each advertisement.

Advertisement Headline Type	Advertisement Image Type					
	Survey-Taking		Family		Soldiers Marching	
	Number (CTR^a^)	Cost (US $)	Number (CTR)	Cost (US $)	Number (CTR)	Cost (US $)
Altruism	18 (0.82)	42.31	9 (1.11)	84.61	26 (1.43)	29.29
Empowerment	4 (0.87)	190.38	16 (0.99)	47.59	25 (1.12)	30.46
Incentive	20 (0.73)	38.08	20 (0.85)	38.08	156 (1.27)	4.88
Sharing	44 (0.97)	17.31	26 (1.06)	29.29	129 (1.64)	5.90
Social Norms	22 (1.10)	34.61	13 (0.99)	58.58	57 (1.81)	13.36

^a^CTR: click-through rate.

There was also a main effect for ad headline on ad engagement outcomes. Specifically, the *sharing* headline was associated with more impressions than the *incentive* (*P*=.045) and *empowerment* (*P*=.004) headlines; more reactions than the *altruism* (*P*=.004) and *empowerment* (*P*=.014) headlines; and higher survey participation than the *social norms* headline (*P*<.001). In addition, the *social norms* headline was associated with higher click-through rates than the *incentive* (*P*<.001), *altruism* (*P*<.001), and *empowerment P*=.001) headlines.

Two of the 15 ad versions generated nearly half (285/587, 49%) of the participants (Table 2). These were the ads containing the image of *soldiers marching* with either the *incentive* or *sharing* headline. These two ad versions had significantly higher impressions (*P*<.001) and CTR (*P*<.001) than the other 13. Consequently, these two ads were most cost effective, at US $4.88 and US $5.90 per participant, respectively. Results were similar when examining individuals who completed the online eligibility screener, regardless of whether they were eligible or completed the full survey.

### Veterans With Suicidal Ideation or Non-Enrolled in Veterans Affairs Health Care

Among veterans who completed the survey, the probability of suicidal ideation ranged from an estimated 15%-50% across the 15 ad variants, and the probability of not being enrolled in VA health care ranged from an estimated 18%-50% (Table 3). Recruitment of veterans with suicidal ideation was significantly higher for ads with the *survey-taking* image, as compared to the *soldiers marching* (*P*=.007) image. There were no statistically significant differences in recruitment of non-enrolled veterans by ad image or headline.

**Table 3:** Predicted probabilities of suicide ideation and non-enrollment in Veterans Affairs Health Care among survey participants.

Advertisement image and headline		Probability of suicidal ideation^a^ (95% CI)	Probability of not being enrolled in veterans affairs health care (95% CI)
**Survey-taking**			
	Altruism	0.28 (0.12-0.52)	0.28 (0.12-0.52)
	Empowerment	0.25 (0.03-0.76)	0.25 (0.03-0.76)
	Incentive	0.45 (0.25-0.66)	0.50 (0.29-0.71)
	Sharing	0.32 (0.20-0.47)	0.30 (0.18-0.44)
	Social norms	0.19 (0.07-0.41)	0.18 (0.07-0.40)
**Family**			
	Altruism	0.22 (0.06-0.58)	0.33 (0.11-0.67)
	Empowerment	0.50 (0.27-0.73)	0.25 (0.10-0.51)
	Incentive	0.20 (0.08-0.43)	0.30 (0.14-0.53)
	Sharing	0.15 (0.06-0.35)	0.38 (0.22-0.58)
	Social norms	0.23 (0.08-0.52)	0.38 (0.17-0.66)
**Soldiers Marching**			
	Altruism	0.19 (0.08-0.39)	0.23 (0.11-0.43)
	Empowerment	0.16 (0.06-0.36)	0.28 (0.14-0.48)
	Incentive	0.17 (0.12-0.24)	0.37 (0.30-0.45)
	Sharing	0.22 (0.15-0.30)	0.33 (0.25-0.41)
	Social norms	0.25 (0.15-0.37)	0.33 (0.22-0.46)

^a^Depressive Symptom Inventory Suicidality Subscale (DSI-SS) score ≥ 2.

## Discussion

### Key Findings

Our study demonstrated that Facebook ads are a potentially powerful tool to recruit research subjects. With the support of a single, half-time research assistant, we engaged veterans in enrollment in our online survey at a rapid clip (ie, nearly 100 participants per week). Though our click-through rate was similar to prior studies, our response rate was very high, which may have reflected ease of participation in this online survey. Average cost per participant was less than US $20, and our best-performing ads were dramatically cheaper, approximately US $5-6 per survey participant, a figure that compares very favorably with most prior studies [37,38]. Facebook ads were further disseminated through social sharing, as is illustrated by the “likes”, comments, and sharing of ads that we observed. This is a significant “externality” from a cost efficiency standpoint.

The feasibility of reaching and engaging younger veterans in research through this approach has important public health and clinical implications. We reached not only a relatively broad target population (ie, recent military veterans) but also were effective in engaging subpopulations that can be hard-to-reach and are of heightened interest. Being able to rapidly reach veterans who are experiencing current suicidal ideation and unengaged in VA health care—as we did—is a major challenge for the VA, health policy-makers and other stakeholders interested in improving veteran mental health outcomes.

It is worth emphasizing the high rate of detection of potentially serious psychiatric problems in this sample; we found high rates of screening positive for active PTSD (266/585, 45%), problematic drinking (243/584, 42%), major depression (164/586, 28%) and serious suicidal ideation (132/585, 23%). Facebook ads, together with other digital media advertising strategies that can support help-seeking (eg, Google AdWords) [39], may comprise critical tools in the design of effective campaigns for mental health treatment engagement. The major—and more imposing—next challenge is how to move individuals from endorsing their distress (online) to engaging in treatment in a health care or other therapeutic setting.

A critical novel component of this study was the use of an experiment, or as close to a “true experiment” as is possible in the Facebook advertising environment, to determine what ad features are most likely to result in engagement with the ad. One of the most intriguing novel findings here was that a headline encouraging users to share the ad resulted in better ad engagement. “Sharing” is a request that is uniquely suited to the social media milieu, and also appeals to a military ethos of helping peers. Results also varied depending on the level of engagement being measured and target population. What works as a “hook” regarding the generating clicks may not translate into more active participation, as was similarly found in a study reaching concerned partners of heavy drinking service members and veterans [19]. We found that for a more modest level of engagement (eg, impressions and clicks) with a broad spectrum of recent military veterans, using an image of soldiers or headlines containing a social norms message may be more effective. In contrast, if the goal is more proactive engagement (ie, survey completion) by individuals with active suicidality, an image of a person taking a survey may work better. One reason careful development and selection of image and text may be necessary to optimize ads for individuals with suicidal ideation could be related to cognitive differences in these individuals [40]. There is, for instance, an emerging set of empirical studies showing attentional biases toward certain words [41,42].

### Limitations and Future Directions

Our results should be considered in the context of several limitations. As participation in this study only involved a one-time online survey, it is not clear if the same strategies would be effective for treatment engagement [39], or engagement in research requiring a higher burden (eg, intervention or longitudinal cohort study). Also, it is possible that individuals perceived our ads in ways different than hypothesized (eg, the “survey-taking” image could have been perceived as that of “computer technology”). Military culture may also impact response to advertisements. For example, our military-related ad images favored the army, which may have contributed a higher representation of them in the sample. Future research focused on testing the effectiveness of online ads should consider a qualitative component to gain more insight into differential ad performance. If future studies can confirm and further identify ad features that result in more response and engagement by veterans with suicidal ideation, there is significant potential for targeted interventions or campaigns to enhance outreach, health messaging, help-seeking, or other behaviors.

### Conclusions

Taken together, our study demonstrates that Facebook ads are an effective medium for rapidly identifying, reaching, and recruiting recent military veterans, and can particularly help in reaching individuals who screen positive for current mental health problems. These results provide a foundation to inform efforts to engage veterans disconnected from the health care system or at risk for suicidal ideation.

## Abbreviations

Ad: advertisements
AUDIT-C: Test Alcohol Consumption Questions
CDA: Career Development Award
CTR: click-through rate
CIVIC: Center to Improve Veteran Involvement in Care
DSI-SS: Depressive Symptom Inventory Suicidality Subscale
OEF-OIF: Operation Enduring Freedom-Operation Iraqi Freedom
OHSU: Oregon Health & Science University
PC-PTSD: Primary Care PTSD Screen for DSM-5
PHQ-2: Patient Health Questionnaire-2
PTSD: posttraumatic stress disorder
US: United States
VA: Veterans Affairs

## References

[1] Seal KH, Bertenthal D, Miner CR, Sen S, Marmar C (2007). Bringing the war back home: mental health disorders among 103,788 US veterans returning from Iraq and Afghanistan seen at Department of Veterans Affairs facilities. Arch Intern Med.

[2] Reger MA, Smolenski DJ, Skopp NA, Metzger-Abamukang MJ, Kang HK, Bullman TA, Perdue S, Gahm GA (2015). Risk of Suicide Among US Military Service Members Following Operation Enduring Freedom or Operation Iraqi Freedom Deployment and Separation From the US Military. JAMA Psychiatry.

[3] Hoffmire CA, Kemp JE, Thompson C (2015). Suicide Prevention in Patient and Nonpatient Populations: In Reply. Psychiatr Serv.

[4] (2017). Analysis of VA Health Care Utilization among Operation Enduring Freedom (OEF), Operation Iraqi Freedom (OIF), and Operation New Dawn (OND) Veterans.

[5] Denneson L, Corson K, Helmer D, Bair M, Dobscha S (2014). Mental health utilization of new-to-care Iraq and Afghanistan Veterans following suicidal ideation assessment. Psych Research.

[6] Gorman LA, Blow AJ, Ames BD, Reed PL (2011). National Guard families after combat: mental health, use of mental health services, and perceived treatment barriers. Psychiatr Serv.

[7] Stecker T, Fortney J, Hamilton F, Sherbourne CD, Ajzen I (2010). Engagement in mental health treatment among veterans returning from Iraq. Patient Prefer Adherence.

[8] Bruffaerts R, Demyttenaere K, Hwang I, Chiu W, Sampson N, Kessler RC, Alonso J, Borges G, de GG, de GR, Florescu S, Gureje O, Hu C, Karam EG, Kawakami N, Kostyuchenko S, Kovess-Masfety V, Lee S, Levinson D, Matschinger H, Posada-Villa J, Sagar R, Scott KM, Stein DJ, Tomov T, Viana MC, Nock MK (2011). Treatment of suicidal people around the world. Br J Psychiatry.

[9] Downs MF, Eisenberg D (2012). Help seeking and treatment use among suicidal college students. J Am Coll Health.

[10] Chartrand H, Robinson J, Bolton JM (2012). A longitudinal population-based study exploring treatment utilization and suicidal ideation and behavior in major depressive disorder. J Affect Disord.

[11] Hynes DM, Koelling K, Stroupe K, Arnold N, Mallin K, Sohn M, Weaver FM, Manheim L, Kok L (2007). Veterans' access to and use of Medicare and Veterans Affairs health care. Med Care.

[12] Basham C, Denneson LM, Millet L, Shen X, Duckart J, Dobscha SK (2011). Characteristics and VA health care utilization of U.S. Veterans who completed suicide in Oregon between 2000 and 2005. Suicide Life Threat Behav.

[13] Kosinski M, Matz SC, Gosling SD, Popov V, Stillwell D (2015). Facebook as a research tool for the social sciences: Opportunities, challenges, ethical considerations, and practical guidelines. Am Psychol.

[14] Stewart J New York Times.

[15] Duggan M (2015). Pew Research Center.

[16] Ramo DE, Hall SM, Prochaska JJ (2010). Reaching young adult smokers through the internet: comparison of three recruitment mechanisms. Nicotine Tob Res.

[17] Ramo DE, Rodriguez TMS, Chavez K, Sommer MJ, Prochaska JJ (2014). Facebook Recruitment of Young Adult Smokers for a Cessation Trial: Methods, Metrics, and Lessons Learned. Internet Interv.

[18] Pedersen ER, Helmuth ED, Marshall GN, Schell TL, PunKay M, Kurz J (2015). Using facebook to recruit young adult veterans: online mental health research. JMIR Res Protoc.

[19] Pedersen E, Osilla K, Helmuth E, Tolpadi A, Gore K (2017). Reaching Concerned Partners of Heavy Drinking Service Members and Veterans through Facebook. Military Behavioral Health.

[20] Platt T, Platt J, Thiel DB, Kardia SLR (2016). Facebook Advertising Across an Engagement Spectrum: A Case Example for Public Health Communication. JMIR Public Health Surveill.

[21] Kramer J, Rubin A, Coster W, Helmuth E, Hermos J, Rosenbloom D, Moed R, Dooley M, Kao Y, Liljenquist K, Brief D, Enggasser J, Keane T, Roy M, Lachowicz M (2014). Strategies to address participant misrepresentation for eligibility in Web-based research. Int J Methods Psychiatr Res.

[22] Dolan P, Hallsworth M, Halpern D, King D, Metcalfe R, Vlaev I (2012). Influencing behaviour: The mindspace way. Journal of Economic Psychology.

[23] Andreoni J (1990). Impure Altruism and Donations to Public Goods: A Theory of Warm-Glow Giving. The Economic Journal.

[24] Avey J, Luthans F, Youssef C (2009). The Additive Value of Positive Psychological Capital in Predicting Work Attitudes and Behaviors. Journal of Management.

[25] Boamah SA, Laschinger H (2016). The influence of areas of worklife fit and work-life interference on burnout and turnover intentions among new graduate nurses. J Nurs Manag.

[26] Cialdini Rb (2007). Descriptive Social Norms as Underappreciated Sources of Social Control. Psychometrika.

[27] Gajic A, Cameron D, Hurley J (2012). The cost-effectiveness of cash versus lottery incentives for a web-based, stated-preference community survey. Eur J Health Econ.

[28] Oeldorf-Hirsch A, Sundar S (2015). Posting, commenting, and tagging: Effects of sharing news stories on Facebook. Computers in Human Behavior.

[29] Appendix A: National Survey of Veterans Questionnaire Instruments.

[30] Greenwood S, Perrin A, Duggan M (2016). Pew Research Center.

[31] Prins A, Bovin MJ, Smolenski DJ, Marx BP, Kimerling R, Jenkins-Guarnieri MA, Kaloupek DG, Schnurr PP, Kaiser AP, Leyva YE, Tiet QQ (2016). The Primary Care PTSD Screen for DSM-5 (PC-PTSD-5): Development and Evaluation Within a Veteran Primary Care Sample. J Gen Intern Med.

[32] Bush K, Kivlahan DR, McDonell MB, Fihn SD, Bradley KA (1998). The AUDIT alcohol consumption questions (AUDIT-C): an effective brief screening test for problem drinking. Ambulatory Care Quality Improvement Project (ACQUIP). Alcohol Use Disorders Identification Test. Arch Intern Med.

[33] Kroenke K, Spitzer RL, Williams JBW (2003). The Patient Health Questionnaire-2: validity of a two-item depression screener. Med Care.

[34] Joiner TE, Pfaff JJ, Acres JG (2002). A brief screening tool for suicidal symptoms in adolescents and young adults in general health settings: reliability and validity data from the Australian National General Practice Youth Suicide Prevention Project. Behav Res Ther.

[35] von GM, Teismann T, Prinz S, Gebauer JE, Hirschfeld G (2016). Depressive Symptom Inventory Suicidality Subscale: Optimal Cut Points for Clinical and Non-Clinical Samples. Clin Psychol Psychother.

[36] Parker K, Cilluffo A, Stepler R (2017). Pew Research Center.

[37] Topolovec-Vranic J, Natarajan K (2016). The Use of Social Media in Recruitment for Medical Research Studies: A Scoping Review. J Med Internet Res.

[38] Whitaker C, Stevelink S, Fear N (2017). The Use of Facebook in Recruiting Participants for Health Research Purposes: A Systematic Review. J Med Internet Res.

[39] Birnbaum ML, Garrett C, Baumel A, Scovel M, Rizvi AF, Muscat W, Kane JM (2017). Using Digital Media Advertising in Early Psychosis Intervention. Psychiatr Serv.

[40] Yiend J (2010). The effects of emotion on attention: A review of attentional processing of emotional information. Cognition & Emotion.

[41] Nock MK, Park JM, Finn CT, Deliberto TL, Dour HJ, Banaji MR (2010). Measuring the suicidal mind: implicit cognition predicts suicidal behavior. Psychol Sci.

[42] Cha CB, Najmi S, Park JM, Finn CT, Nock MK (2010). Attentional bias toward suicide-related stimuli predicts suicidal behavior. J Abnorm Psychol.

