# Correction: Reaching Those At Risk for Psychiatric Disorders and Suicidal Ideation: Facebook Advertisements to Recruit Military Veterans

**DOI:** 10.2196/13035

**Published:** 2019-01-09

**Authors:** Alan R Teo, Samuel BL Liebow, Benjamin Chan, Steven K Dobscha, Amanda L Graham

**Affiliations:** 1 HSR&D Center to Improve Veteran Involvement in Care (CIVIC) VA Portland Health Care System Portland, OR United States; 2 Department of Psychiatry Oregon Health & Science University Portland, OR United States; 3 School of Public Health Oregon Health & Science University and Portland State University Portland, OR United States; 4 Schroeder Institute for Tobacco Research & Policy Studies Truth Initiative Washington, DC United States; 5 Department of Oncology Georgetown University Medical Center Washington, DC United States

The authors of “Researching Those At Risk for Psychiatric Disorders and Suicidal Ideation: Facebook Advertisements to Recruit Military Veterans” (JMIR Ment Health 2018;5(3):e10078) incorrectly labeled some column headers in Table 2. Currently, the columns list the click-through rate as a percentage (“CTR^a^, n (%)”). They should be labeled as simply the number and click-through rate (“Number (CTR^a^)”).

The correction will appear in the online version of the paper on the JMIR website on January 9, 2019, together with the publication of this correction notice. Because this was made after submission to PubMed, PubMed Central, and other full-text repositories, the corrected article also has been resubmitted to those repositories.

